# Newer versus Early Generation of the MitraClip for Primary Mitral Regurgitation: A Japanese Single-Center Experience

**DOI:** 10.31083/j.rcm2405138

**Published:** 2023-05-05

**Authors:** Taishi Okuno, Masaki Izumo, Noriko Shiokawa, Shingo Kuwata, Yuki Ishibashi, Yukio Sato, Masashi Koga, Kazuaki Okuyama, Norio Suzuki, Keisuke Kida, Yasuhiro Tanabe, Yoshihiro J Akashi

**Affiliations:** ^1^Department of Cardiology, St. Marianna University Hospital, 216-8511 Kawasaki, Japan; ^2^Department of Pharmacology, St. Marianna University School of Medicine, 216-8511 Kawasaki, Japan

**Keywords:** transcatheter edge-to-edge repair, mitral valve repair, MitraClip, primary mitral regurgitation

## Abstract

**Background:**

The MitraClip G4 system is the latest version of the transcatheter edge-to-edge repair (TEER) system for mitral regurgitation (MR). We aimed to investigate the impact of the new system on routine clinical practice and patient outcomes in the treatment of primary MR.

**Methods:**

Consecutive patients with primary MR who underwent TEER with either the MitraClip G2 or G4 between 2018 and 2021 were enrolled from a single center registry. Baseline clinical and echocardiographic characteristics as well as procedural and clinical outcomes up to 1 year were compared between groups. Technical and device success were defined in accordance with the Mitral Valve Academic Research Consortium criteria.

**Results:**

Among 71 patients with primary MR, 34 were treated with G2 and 37 were treated with G4. Patients treated with G4 had lower surgical risk (7.74 [5.04, 14.97] vs. 5.26 [3.98, 6.40]; *p <* 0.01) than those with G2. There were no significant differences in other baseline clinical variables between groups. On baseline echocardiography, MR volume and flail gap were significantly greater in the G4 group than in the G2 group (regurgitant volume: 63 [41–76] mL vs. 68 [62–84] mL; *p* = 0.04, flail gap: 4.5 [3.5–5.5] mm vs. 5.4 [4.5–7.1] mm; *p* = 0.04). Technical success was achieved in over 95% of both groups with no significant difference (*p *> 0.99). Device success was achieved in 61.8% of the G2 group, while in 70.3% of the G4 group (*p* = 0.47). Post-procedural MR severity was comparable (*p* = 0.42) and there was no significant difference in the occurrence of mitral stenosis (*p* = 0.61) between groups. Among patients who reached 1-year follow-up (n = 54), there was no significant difference between groups in a composite endpoint of death or heart failure rehospitalization (10.5% vs. 20.2%; HR 0.61; 95% CI 0.17–2.22; *p* = 0.45). Residual heart failure symptoms (NYHA ≥3) at 1 year were observed in 3.7% of the G2 group, while no patient in the G4 group (*p *> 0.99).

**Conclusions:**

The MitraClip G4 system achieved comparable device outcomes to the early-generation device (G2), despite treating more severe primary MR with a larger flail gap.

## 1. Introduction

Transcatheter edge-to-edge repair (TEER) has become an established therapeutic alternative to mitral valve surgery for patients with severe primary mitral regurgitation (MR) and high or prohibitive surgical risk [1, 2, 3]. Since the regulatory approval of the MitraClip mitral valve repair system (Abbott Vascular, Abbott Park, IL, USA) as the first TEER device in Europe in 2008, in the United States in 2013, and in Japan in 2018, challenging mitral valve anatomy for the system has been identified, and iterative refinements have been made to the device and delivery system [4]. The MitraClip G4 system is the newest iteration and is currently being used worldwide [5, 6]. The EXPAND G4 study (NCT04177394), a post-market, multicenter, single-arm, prospective study, is ongoing and will report the safety and performance of the MitraClip G4 system. However, the impact of the new system on current clinical practice has not been well studied. Therefore, our study aimed to investigate the impact of the introduction of the new MitraClip system on routine clinical practice and patient outcomes up to 1 year in the treatment of primary MR in a Japanese single-center prospective registry.

## 2. Methods

### 2.1 Study Population

We consecutively enrolled all patients who underwent TEER with the MitraClip mitral valve repair system at St. Marianna University Hospital in a prospective registry. The registry is part of a multicenter registry approved by the local institutional review board (No. 4209) and registered with the University Hospital Medical Information Network (Treatment and prognosis of heart valve registry, UMIN-ID: 000023653). All patients provided written informed consent to participate in the registry, and the study was conducted in accordance with the Declaration of Helsinki.

For the purpose of the present study, patients with primary MR who underwent TEER either with the MitraClip G2 or G4 between 2018 and 2021 were included and retrospectively analyzed.

### 2.2 Procedure

All procedures were discussed and planned by the Heart Team in accordance with established best practice guidelines [3]. Prior to the procedure, a standardized transthoracic echocardiography was performed by an echocardiography specialist. The procedures were performed under general anesthesia with the guidance of two- and three-dimensional transesophageal echocardiography and fluoroscopy in a hybrid operating room. The MitraClip G4 system has been available at our center since October 2020, and it offers four different clip sizes and allows for independent grasping of the anterior and posterior mitral valve leaflets. The selection of clip size was based on careful anatomical assessment of the mitral valve using intraprocedural transesophageal echocardiography. Transthoracic echocardiography was performed on day 3 after the procedure or at the latest, before hospital discharge.

### 2.3 Data Collection and Definitions

Clinical, echocardiographic, procedural, and follow-up data were prospectively collected in an institutional integrated data system. Regular clinical follow-up was scheduled at 30 days, at 1 year, and yearly thereafter. Clinical follow-up data were obtained through documentation from referring physicians, hospital discharge summaries, and standardized telephone interviews. Technical success and device success were retrospectively adjudicated by experienced cardiologists based on the Mitral Valve Academic Research Consortium (MVARC) criteria [7, 8]. Technical success included the following criteria: (1) absence of procedural mortality; (2) successful access, delivery, and retrieval of the device delivery system; (3) successful deployment and correct positioning of the first intended device; and (4) freedom from emergency surgery or reintervention related to the device or access procedure. Device success included the following criteria: (1) absence of procedural mortality or stroke; (2) proper placement and positioning of the device; (3) freedom from unplanned surgical or interventional procedures related to the device or access procedure; and (4) continued intended safety and performance of the device. Intended safety and performance of the device was included: (a) no evidence of structural or functional failure; (b) no specific device-related technical failure issues and complications; (c) and reduction of MR to either optimal or acceptable levels (reduction by at least 1 class/grade from baseline and to no more than 2+ in severity) without significant mitral stenosis (post-procedure effective orifice area is ≥1.5 ${\mathrm{mm}}^{2}$ with a transmitral gradient <5 mmHg). Mitral valve effective orifice area was measured by using the planimetry method. Mitral regurgitation was graded as 0, 1+, 2+, 3+, 4+ according to the MVARC criteria. Optimal mitral valve anatomy for TEER was defined as having: (1) a central jet (A2/P2), (2) a mitral valve area >4 ${\mathrm{cm}}^{2}$, (3) a posterior leaflet length >10 mm, (4) a flail gap <10 mm, and (5) flail width <15 mm [1].

### 2.4 Statistical Analysis

Categorical data are represented as frequencies and percentages and the differences between groups are evaluated with the Chi-square test or Fisher’s exact test. Continuous variables are expressed as median values and interquartile ranges (IQR) and compared between groups using Mann-Whitney’s U test. Event-free survival curves were constructed using the Kaplan-Meier method and Cox proportional hazards models were used to calculate hazard ratios (HR) and 95% confidence intervals (95% CI). Throughout the present study, a *p*-value of <0.05 was considered significant. Statistical analyses were performed using EZR software 1.61 (Saitama Medical Center, Jichi Medical University, Saitama, Japan) which is a graphical user interface for R 4.2.1 (The R Foundation for Statistical Computing, Vienna, Austria).

## 3. Results

### 3.1 Patients

During the study period, 223 patients underwent TEER with the MitraClip mitral valve repair system at our center. Among them, 71 patients with primary MR who met the inclusion criteria were retrospectively analyzed. Of these, 34 patients were treated with the G2 system and 37 patients with the G4 system. Baseline characteristics are summarized in Table 1. There were no significant differences in baseline clinical characteristics, except for a higher surgical risk in the G2 group than in the G4 group (Society of Thoracic Surgeons Predicted Risk of Mortality: 7.74 [5.04–14.97], G2 vs. 5.26 [3.98–6.40], G4; *p <*0.01).

**Table 1.** **Baseline characteristics**.

	G2	G4	*p*-value
	N = 34	N = 37	
Age (years)	83 [76–86]	85 [81–88]	0.18
Sex (male)	19 (55.9%)	23 (62.2%)	0.64
Body mass index (kg/${\mathrm{cm}}^{2}$)	20.9 [19.0–23.3]	21.4 [18.4–23.8]	0.68
STS PROM	7.74 [5.07–14.97]	5.26 [3.98–6.40]	<0.01
NYHA III or IV	24 (70.6%)	27 (73.0%)	>0.99
Hypertension	26 (76.5%)	26 (70.3%)	0.60
Diabetes mellitus	7 (20.6%)	5 (13.5%)	0.53
Chronic kidney disease (eGFR <60)	24 (70.6%)	24 (66.7%)	0.80
Atrial fibrillation	18 (56.2%)	14 (41.2%)	0.32
Preserved LVEF (≥50%)	33 (97.1%)	34 (91.9%)	0.62
Pulmonary hypertension (SPAP ≥40 mmHg)	11 (32.4%)	8 (21.6%)	0.42

STS PROM, Society of Thoracic Surgeons Predicted Risk of Mortality; NYHA, New York Heart Association; eGFR, estimated glomerular filtration rate; LVEF, left ventricular ejection fraction; SPAP, systolic pulmonary artery pressure.

Baseline echocardiographic data are detailed in Table 2. The volume of mitral regurgitation was greater in the G4 group than in the G2 group (regurgitant volume: 63 [41–76] mL, G2 vs. 68 [62–84] mL, G4; *p* = 0.04; effective regurgitant orifice area: 0.43 [0.30–0.49] ${\mathrm{cm}}^{2}$, G2 vs. 0.47 [0.41–0.58] ${\mathrm{cm}}^{2}$, G4; *p* = 0.07). The flail gap was significantly greater in the G4 group than in the G2 group (4.5 [3.5–5.5] mm, G2 vs. 5.4 [4.5–7.1] mm, G4; *p* = 0.04), while flail width was comparable between the two groups (9.1 [7.6–10.7] mm, G2 vs. 10.2 [8.1–12.3] mm, G4; *p* = 0.18). Mitral valve area by planimetry was significantly greater in the G4 group than in the G2 group (4.9 [4.4–5.7] ${\mathrm{cm}}^{2}$, G2 vs. 5.9 [5.0–6.6] ${\mathrm{cm}}^{2}$, G4; *p* = 0.01), while mean transmitral gradient was comparable between the two groups (2.0 [1.3–2.3] mmHg, G2 vs. 1.7 [1.4–2.1] mmHg, G4; *p* = 0.98). There was no significant difference in the length of posterior leaflet (11.2 [9.3–13.0] mm, G2 vs. 11.0 [9.5–12.0] mm, G4; *p* = 0.81). The mitral valve anatomy was considered optimal for TEER in 52.9% of the G2 group, while in 38.3% of the G4 group (*p* = 0.20). The other echocardiographic parameters including left ventricular (LV) systolic function, LV dimensions, prevalence of moderate or greater tricuspid regurgitation, tricuspid annular plane systolic excursion (TAPSE), and systolic pulmonary artery pressure (SPAP), were comparable between the two groups.

**Table 2.** **Echocardiographic characteristics**.

	G2	G4	*p*-value
	N = 34	N = 37	
LVEF (%)	64.5 [59.3–71.8]	66.0 [59.0–69.0]	0.80
LVEDV (mL)	100.0 [83.8–126.3]	101 [91.0–134.0]	0.57
LVESV (mL)	35.0 [26.5–46.0]	36.0 [26.0–52.0]	0.73
Regurgitant volume (mL)	63 [41–76]	68 [62–84]	0.04
EROA (${\mathrm{cm}}^{2}$)	0.43 [0.30–0.49]	0.47 [0.41–0.58]	0.07
Pathology in A2-P2 zone	22 (64.7%)	21 (58.3%)	0.63
Posterior leaflet length, mm	11.2 [9.3–13.0]	11.0 [9.5–12.0]	0.81
flail gap, mm	4.5 [3.5–5.5]	5.4 [4.5–7.1]	0.04
flail width, mm	9.1 [7.6–10.7]	10.2 [8.1–12.3]	0.18
Mean pressure gradient, mmHg	2.0 [1.3–2.3]	1.7 [1.4–2.1]	0.98
Mitral valve area, ${\mathrm{cm}}^{2}$	4.9 [4.4–5.7]	5.9 [5.0–6.6]	0.01
Optimal mitral valve anatomy for MitraClip*	18 (52.9%)	23 (38.3%)	0.20
Moderate or severe TR	10 (29.4%)	11 (29.7%)	>0.99
TAPSE, mm	18.2 [12.7–22.5]	19.3 [18.0–20.5]	0.42
SPAP, mmHg	32.3 [24.7–44.2]	32.8 [25.7–38.8]	0.90

LVEF, left ventricular ejection fraction; LVESV, left ventricular end-systolic volume; LVEDV, left ventricular end-diastolic volume; EROA, effective regurgitant orifice area; TR, tricuspid regurgitation; TAPSE, tricuspid annular plane systolic excursion; SPAP, systolic pulmonary artery pressure. *Optimal mitral valve anatomy for TEER was defined as having: (1) a central jet (A2/P2), (2) a mitral valve area >4 ${\mathrm{cm}}^{2}$, (3) a posterior leaflet length >10 mm, (4) a flail gap <10 mm, and (5) flail width <15 mm.

### 3.2 Procedural Characteristics and Outcomes

**Fig. 1.**
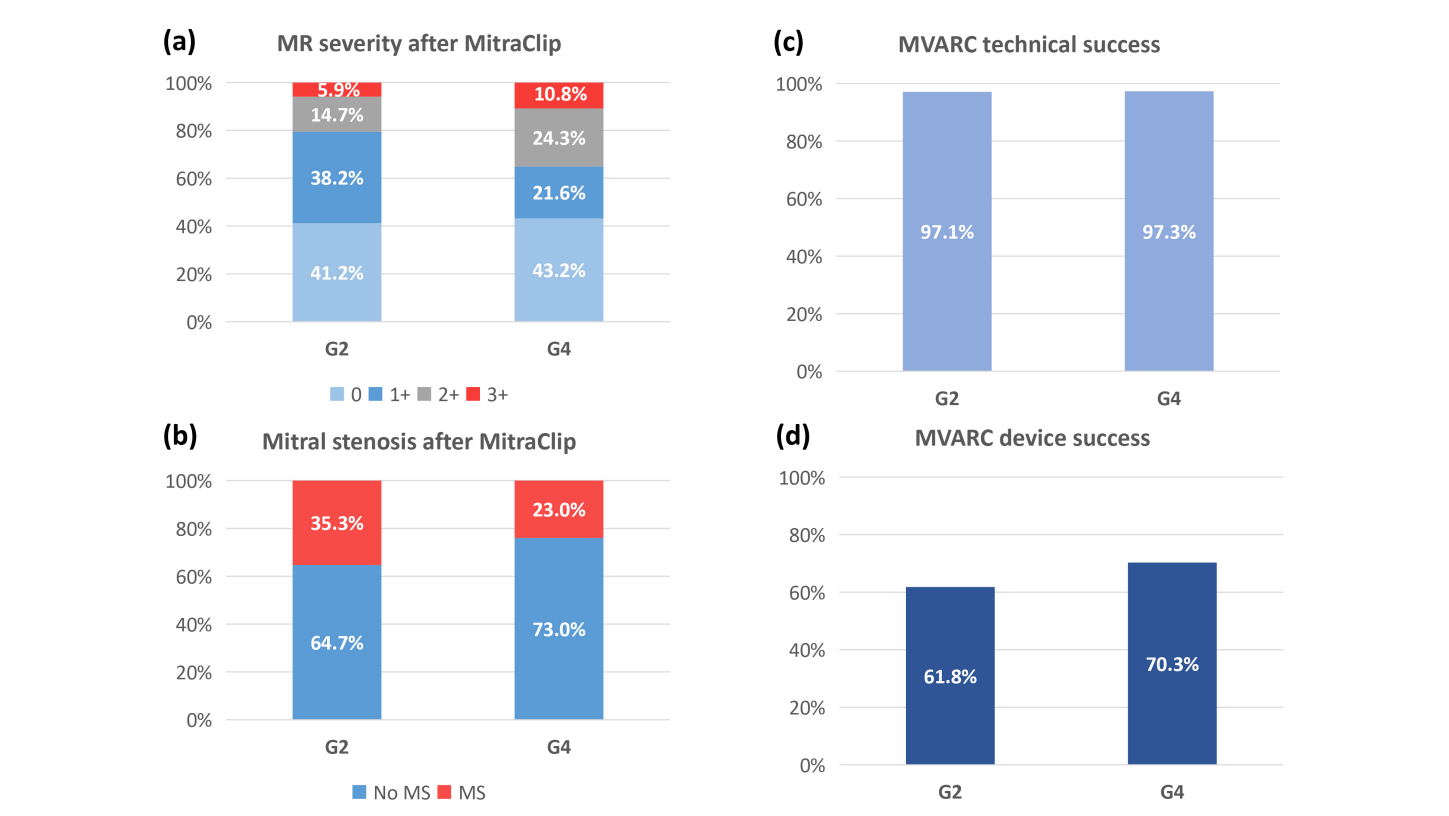
**Procedural outcomes of the G2 versus G4 MitraClip system**. (a) Acceptable level of MR reduction (≤2+) was achieved in approximately 90% of patients without difference between the two groups (5.9% vs. 10.8%; *p* = 0.68). (b) There was no significant difference in the occurrence of mitral stenosis (post-procedure effective orifice area is <1.5 ${\mathrm{mm}}^{2}$ with a transmitral gradient ≥5 mmHg) after MitraClip between the two groups (35.3% vs. 27.0%; *p* = 0.61). (c) MVARC technical success was achieved in more than 95% of patients without difference between groups (97.1% vs. 97.3%; *p *> 0.99). (d) MVARC device success was achieved in 61.8% in the G2 group, and in 70.3% in the G4 group, with no significant difference between the two groups (*p* = 0.47). MR, mitral regurgitation; MS, mitral stenosis; MVARC, Mitral Valve Academic Research Consortium.

Procedural characteristics and outcomes are shown in Table 3. No significant differences were observed in the median procedural time and the number of clips used. In the G4 group, 54.1% of patients were treated with at least one extended arm clip and 31 out of 37 patients (83.8%) received at least one wide clip (NTW or XTR). Procedural complications were rare in both groups; single leaflet device attachment occurred in one case in the G4 group, and emergency surgery related to the device occurred in one case in the G2 group. MVARC technical success was achieved in more than 95% of patients without a difference between the two groups (97.1%, G2 vs. 97.3%, G4; *p *> 0.99) (Fig. 1).

**Table 3.** **Procedural characteristics and outcomes**.

			G2	G4	*p*-value
			N = 34	N = 37	
Procedural time, min			81 [63–127]	81 [66–116]	0.96
Number of clips					0.16
	1		16 (47.1%)	24 (64.9%)	
	2		18 (52.9%)	13 (35.1%)	
Number of clips			2 [1–2]	1 [1–1]	0.14
Extended arm clips (XT/XTW)			NA	20 (54.1%)	NA
Wide clips (NTW/XTW)			NA	31 (83.8%)	NA
Technical Success			33 (97.1%)	36 (97.3%)	>0.99
	Procedural death		0 (0%)	0 (0%)	NA
	Deployment failure		0 (0%)	0 (0%)	NA
	SLDA		0 (0%)	1 (2.7%)	>0.99
	Emergency surgery/intervention related to the procedure		1 (2.9%)	0 (0%)	0.48
Device Success			21 (61.8%)	26 (70.3%)	0.47
Echocardiographic outcome					
	Residual MR >2+		2 (5.9%)	4 (10.8%)	0.68
	MR grade				0.42
		0	14 (41.2%)	16 (43.2%)	
		1+	13 (38.2%)	8 (21.6%)	
		2+	5 (14.7%)	9 (24.3%)	
		3+	2 (5.9%)	4 (10.8%)	
	Mean transmitral gradient, mmHg		2.9 [2.1–4.1]	2.3 [1.7–3.7]	0.10
	Mean transmitral gradient >5 mmHg		3 (8.8%)	4 (10.8%)	>0.99
	MVA (planimetry), ${\mathrm{cm}}^{2}$		1.85 [1.45–2.74]	2.11 [1.61–2.78]	0.60
	MVA (planimetry) <1.5 ${\mathrm{cm}}^{2}$		10 (29.4%)	8 (22.2%)	0.59
	Mitral Stenosis		12 (35.3%)	10 (27.0%)	0.61

SLDA, single leaflet device attachment; MR, mitral regurgitation; MVA, mitral valve area.

Echocardiographic data at discharge are also shown in Table 3. After MitraClip, an acceptable level of MR reduction (≤2+) was achieved in approximately 90% of patients without a difference between the two groups (5.9%, G2 vs. 10.8%, G4; *p* = 0.68) (Fig. 1). There was no significant difference in the occurrence of mitral stenosis (post-procedure effective orifice area is <1.5 ${\mathrm{mm}}^{2}$ with a transmitral gradient ≥5 mmHg) after MitraClip between the two groups (35.3%, G2 vs. 27.0%, G4; *p* = 0.61) (Fig. 1). MVARC device success was achieved in 61.8% of the G2 group and in 70.3% of the G4 group, with no significant difference between the two groups (*p* = 0.47) (Fig. 1).

### 3.3 Clinical Outcomes

Clinical outcomes were assessed in all patients in the G2 group and in 20 out of 37 patients in the G4 group who reached 1-year clinical follow-up. At 30 days, there were no deaths in either group. Residual heart failure symptoms (NYHA ≥3) were observed in 6.5% in the G2 group, while no patients in the G4 group had residual heart failure symptoms (*p* = 0.22). At 1 year, the composite endpoint of all-cause death and heart failure rehospitalization occurred in 2 patients in the G2 group and in 1 patient in the G4 group (HR 0.85; 95% CI 0.08–9.35; *p* = 0.89) (Table 4). Residual heart failure symptoms (NYHA ≥3) at 1 year were observed in 3.7% of the G2 group, while no patients in the G4 group had residual heart failure symptoms (*p >* 0.99).

**Table 4.** **Clinical outcomes**.

		G2	G4	HR (95% CI)	*p*-value
		N = 34	N = 37		
At 30 days					
	All-cause death	0 (0%)	0 (0%)	NA	NA
	NYHA III or IV	2/31 (6.5%)	0/35 (0%)	NA	0.22
At 1 year		N = 34	N = 20		
Composite endpoint (death of HF rehospitalization)		2 (5.9%)	1 (5.0%)	0.85 (0.08–9.35)	0.89
	All-cause mortality	1 (2.9%)	1 (5.0%)	1.74 (0.11–27.9)	0.69
	HF rehospitalization	1 (2.9%)	0 (0%)	NA	>0.99
	NYHA III or IV	1/27 (3.7%)	0/16 (0%)	NA	>0.99

NYHA, New York Heart Association; HF, heart failure; NA, not assessable.

## 4. Discussion

In this preliminary study, which was based on a small, single-center cohort, we observed the following:

(1) Although the baseline clinical demographics were similar between patients who received the early and newer generations of the MitraClip system, those who were treated with the newer-generation MitraClip system had a lower average surgical risk.

(2) Conversely, patients treated with the newer-generation MitraClip system had more severe MR with a larger flail gap than those with the early-generation system.

(3) Procedural complications were rare in both generations, and the rates of MVARC technical success and device success were comparable between the early- and newer-generation MitraClip systems.

(4) The incidences of all-cause death and heart failure rehospitalization were low, and most patients experienced an improvement in NYHA functional class at 1 year, regardless of the generations of the MitraClip system.

The lower average surgical risk in the G4 group compared to the G2 group suggests that a broader spectrum of patients with primary MR is being treated with TEER along with the maturity of the treatment and advancement of the device over time. In contrast to the surgical risk, the severity of MR is greater and the flail gap is larger in the G4 group than in the G2 group. Importantly, despite the increased severity of baseline MR and the larger flail gap in the G4 group, the G4 devices yielded comparable technical and device success rates compared to the G2 device in the present study. Furthermore, as in the G2 group, most patients benefited from improved heart failure symptoms at 1 year, and event rates, in terms of all-cause death and heart failure rehospitalization, were low through 1-year follow-up.

The Endovascular Valve Edge-to-Edge Repair Study II (EVEREST II) was the first randomized controlled trial to compare TEER with mitral valve surgery in patients with moderately severe and severe primary MR [9]. In brief, TEER was found to be less effective in reducing MR and was associated with an increased risk of subsequent surgery for mitral valve dysfunction within 6 months compared to conventional surgery. Nevertheless, there was no difference in survival up to 5 years after TEER and surgery [10]. Furthermore, TEER was superior in terms of safety with fewer major adverse events than surgery, and both treatments were associated with sustained improvements in heart failure symptoms and left ventricular dimensions through 5-year follow-up. These findings have been confirmed by subsequent registry studies [11, 12, 13] and form the basis for current guideline recommendations that TEER is a reasonable treatment option for patients with severe primary MR who are at high- or prohibitive surgical risk patients [1, 2, 3].

Of note, the EVEREST II trial was conducted between 2005 and 2008, during when patients were treated with the early generation of the MitraClip system. Thereafter, the outcomes of TEER have significantly improved owing to the advancements in techniques and the accumulation of experiences [11, 12, 13, 14]. Indeed, in a recent registry-based study, only 4.7% of patients had procedural complications, and residual MR greater than moderate was observed in only 7.6% of patients [14]. Consistent with these recent registry studies [11, 12, 13, 14], the short- and mid-term outcomes of TEER with the early-generation device in the present study seem improved, in terms of the reduction of MR and re-intervention rate, compared with those in the EVEREST II trial.

The newer-generation MitraClip G4 system offers several advantages over the early-generation device, including the ability to select the optimal clip size from four different sizes based on the individual mitral valve anatomy and independent grasping. These features may potentially lead to improved procedural and clinical outcomes following TEER. Although the present study did not demonstrate a significant improvement in short- and mid-term outcomes with the newer-generation devices compared to the early-generation device, it is noteworthy that the newer-generation devices were able to achieve comparable outcomes to the early-generation device despite treating patients with more severe MR with a larger flail gap. This highlights the effectiveness and versatility of these newer-generation devices. Further studies are needed to assess the impact of device evolution on procedural and clinical outcomes following TEER, as well as an optimal patient selection for the treatment of primary MR.

### Study Limitations

The results of the present analysis need to be interpreted in light of several important limitations. First, the study population in the present analysis was small, which may have been insufficient to detect small differences in procedural and clinical outcomes between devices. The low event rate of rare procedural complications and clinical outcomes warrants cautious interpretation. In turn, the robustness of the findings on device outcomes, including MVARC technical and device success, was reinforced by the independent event adjudication based on detailed documentation of endpoints prospectively collected in the registry. Second, this was a before-and-after study by its nature and is subject to bias due to temporal changes in clinical practice, as well as the effect of the learning curve of the procedure. The small number of patients did not allow us to adequately adjust for confounding factors. Lastly, the results of the present study reflect the experience of a single high-volume center, and the results may not be generalizable to other centers. Thus, the findings need to be corroborated by larger, multicenter studies.

## 5. Conclusions

Since the introduction of the newer generation of the MitraClip system, a broader spectrum of patients with primary MR are being treated with TEER, in terms of surgical risk and MR severity. The newer-generation devices achieved comparable device outcomes to the early-generation device, despite treating more severe primary MR with a larger flail gap.

## Data Availability

Data will be shared on request to the corresponding author with permission of St. Marianna University Hospital.
